# SIRT3 inhibits prostate cancer by destabilizing oncoprotein c-MYC through regulation of the PI3K/Akt pathway

**DOI:** 10.18632/oncotarget.4764

**Published:** 2015-07-03

**Authors:** Yizhou Quan, Naitao Wang, Qianqian Chen, Jin Xu, Wei Cheng, Meijuan Di, Weiliang Xia, Wei-Qiang Gao

**Affiliations:** ^1^ State Key Laboratory of Oncogenes and Related Genes, Renji-MedX Clinical Stem Cell Research Center, Ren Ji Hospital, School of Biomedical Engineering, Shanghai Jiao Tong University, Shanghai, China; ^2^ Department of Urology, First People's Hospital of Xiaoshan, Hangzhou, Zhejiang, China; ^3^ Department of Pathology, First People's Hospital of Xiaoshan, Hangzhou, Zhejiang, China; ^4^ Collaborative Innovation Center of Systems Biomedicine, Shanghai, China

**Keywords:** SIRT3, prostate cancer, oncoprotein, PI3K-Akt pathway, c-MYC

## Abstract

SIRT3 is involved in aging-related diseases including cancer, but its role in prostate cancer and detailed regulatory function are not known. We found that SIRT3 was moderately down-regulated in prostate carcinomas. Overexpression of SIRT3 by lentiviral transfection inhibited prostate cancer growth both *in vitro* and *in vivo*, whereas knockdown of SIRT3 increased prostate tumor growth. Mechanistically, the tumor suppression effect of SIRT3 was achieved via its inhibition of the PI3K/Akt pathway. Notably, upregulation of SIRT3 suppressed the phosphorylation of Akt, leading to the ubiquitination and degradation of oncoprotein c-MYC; this could be attenuated by constitutive activation of PI3K/Akt signaling. Collectively, our results unveiled SIRT3's tumor suppressive function and the underlying mechanism in prostate cancer, which might provide therapeutic implications for the disease.

## INTRODUCTION

Prostate cancer is the most common, and second most deadly cancer type for US men [1]. Its occurrence in developing countries including China is also rapidly rising [2]. Patients are likely to relapse after primary therapy [3], and have limited treatment options. Uncontrolled progression of prostate cancer poses a critical challenge in the clinic [4, 5], and clearly it is needed to elucidate the mechanisms of prostate cancer progression and find new therapeutic targets.

Oncogenic genes such as c-MYC that are expressed at high levels in many types of cancers are tumor-inducing factors that prevent programmed cell death and cause uncontrolled cell proliferation. Strategies towards the inhibition of oncogenic genes expressions are actively pursued [6–8]. However, oncoproteins like c-MYC are not readily druggable. Hence, finding new ways and understanding the underlying mechanism of inhibiting oncogenic genes is urgently needed.

Mainly located in the mitochondrion, SIRT3 belongs to the Sirtuin family and is a longevity protein that could extend lifespan by suppressing oxidative stress [9]. Thus far antioxidant therapies for cancer patients have not been successful because antioxidants could hardly access to the mitochondria-localized pools of ROS [10, 11]. In this regard, therapies that directly suppress mitochondria-derived ROS could be an ideal approach [10]. Initially identified as a tumor suppressor in breast cancer, SIRT3 maintained the integrity of mitochondria during stress and Hif1α destabilization [12, 13]. However, little is known about SIRT3's function in prostate cancer. In this study, we report that SIRT3 could act as a prostate tumor suppressor through inhibition of the PI3K/Akt pathway, resulting in ubiquitination and degradation of oncoprotein c-MYC. Up-regulation of SIRT3 might serve as new therapeutic strategies for prostate cancer.

## RESULTS

### SIRT3 is moderately down-regulated in human prostate carcinoma and positively correlates with patient survival

**Figure 1:**
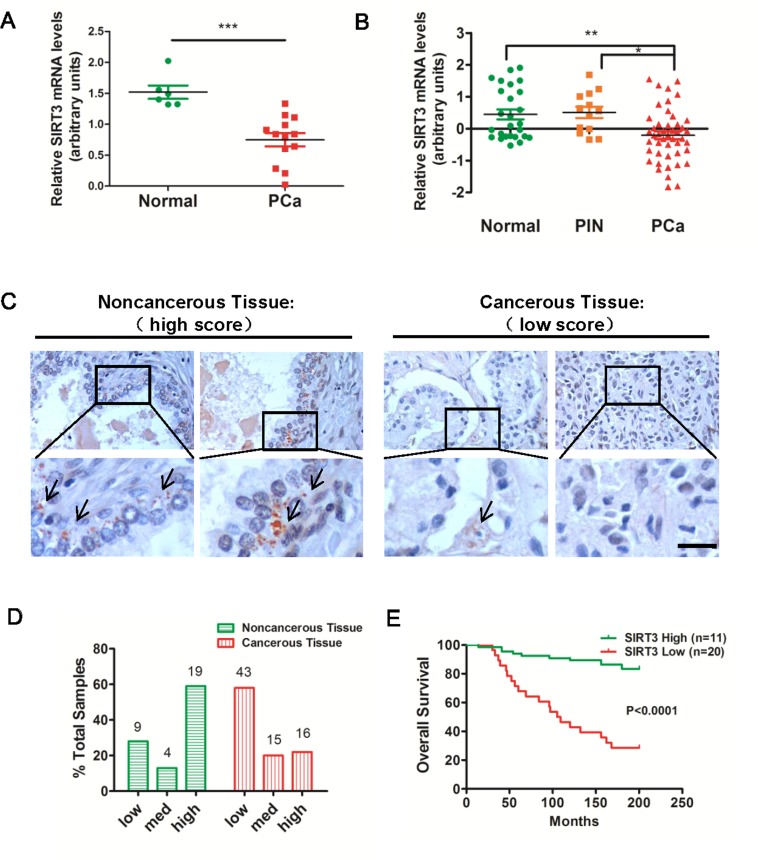
SIRT3 is down-regulated in human prostate carcinoma (**A**) Relative mRNA expression levels of SIRT3 in normal prostate gland and prostate carcinoma (PCa) samples from selected *Oncomine* database (Varambally Prostate Statistics, n=19, ***p < 0.001, Student's t-test). (**B**) Relative mRNA expression levels of SIRT3 in normal, prostatic intraepithelial neoplasia (PIN), benign prostatic hyperplasia (BPH) and prostate carcinoma (PCa) samples from selected *Oncomine* database (Tomlin Prostate Statistics, n=96, *p < 0.05; **p < 0.01, One-way ANOVA followed by Tukey's multiple comparison test). (**C**) Representative immunohistochemical staining of SIRT3 on noncancerous and cancerous human prostate tissue paraffin sections (n=109). Bar =50 μm. (**D**) Percentage of SIRT3 high, medium and low staining scores in non- and carcinoma samples. See also Supplementary Table 1. (**E**) Probabilities of overall survival (%) in total 31 prostate cancer patients from *Oncomine* database were analyzed by Kaplan-Meier survival analysis (log-rank test) comparing high and low level of SIRT3 copy number (Grasso Prostate Statistics). Data are expressed as means ±SEM for (A), (B).

To explore the relationship between SIRT3 expression levels and prostate cancer progression, we first examined multiple microarray datasets in the *Oncomine* Database (www.oncomine.com). Analysis of a dataset containing 19 human clinical specimens showed that SIRT3 mRNA level in prostate carcinoma (PCa) was much lower than that in normal prostate tissues (Figure 1A). A second dataset including 96 human samples also revealed down-regulation of SIRT3 mRNA in PCa relative to normal tissue or prostatic intraepithelial neoplasia (PIN) (Figure 1B). To provide additional evidence in support of this notion, we performed immunohistochemical (IHC) staining of SIRT3 in primary tumors of prostate cancer patients (n=109, of which 32 biopsies were benign tissues, and 77 biopsies were tumor tissues). IHC microscopy analysis revealed that most of SIRT3 signal was localized to epithelial cells in noncancerous tissues (Figure 1C). Samples were scored based on the percentage of cytoplasmic stained, SIRT3-positive cells, and were arbitrarily divided into low (<10% positive), medium (10%-50% positive) and high (>50% positive) groups. In noncancerous samples the majority (56%, 19/32) was SIRT3-high scored, whereas in carcinoma samples, the percentage of SIRT3-high scored plummeted to 22% (16/74) with the SIRT3-low scored rising up to 58% (43/74) (Figure 1D). A detailed description of the clinical features of the patient samples and the levels of SIRT3 staining was provided in Supplementary Table 1. These results corroborated Oncomine data analyses, and further supported a downregulation of SIRT3 expression in prostate cancer samples.

To determine whether SIRT3 expression was associated with patients' survival, we examined multiple microarray data sets from *Oncomine* and performed Kaplan-Meier's survival analysis. The gene copy number of SIRT3 in prostate cancer samples differentiated patients' prognosis, with the high copy number group showing significantly longer overall survival than the other group (Figure 1E). Taken together, these results implicated that SIRT3 was negatively correlated with clinical outcome of prostate cancer patients.

### Overexpression of SIRT3 inhibits prostate cancer cell proliferation *in vitro* and *in vivo*

**Figure 2:**
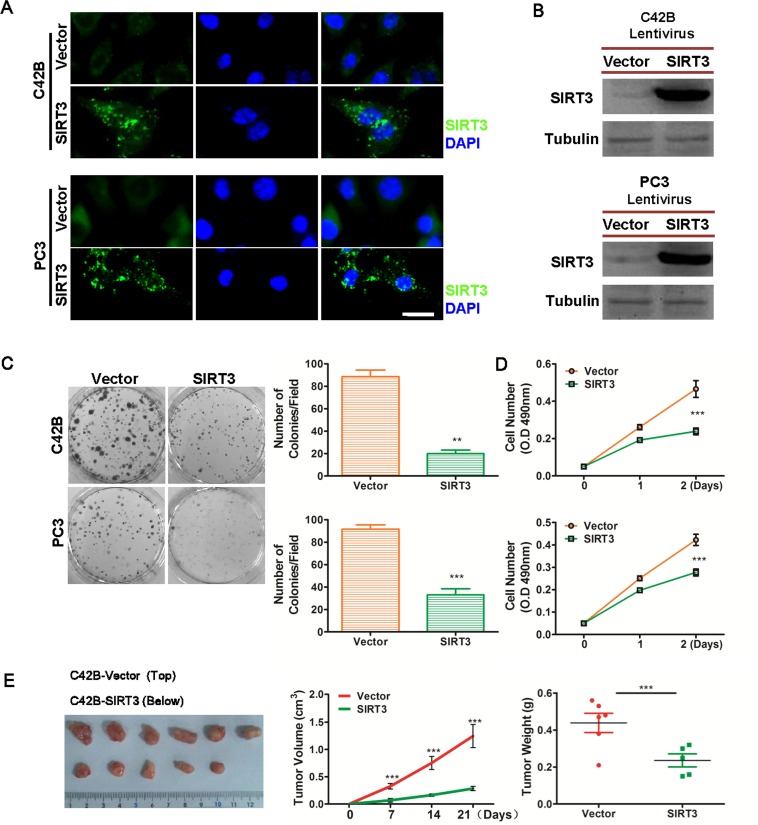
Overexpression of SIRT3 suppresses prostate cancer cell growth Immunofluorescence microscopy (**A**) and Western blot (**B**) analysis of SIRT3 expression in stably overexpressed (SIRT3) and control (Vector) cells (C42B and PC3 PCa cell lines). Bar =20 μm. (**C**) Clone formation assay of SIRT3 stably overexpressed (SIRT3) and control (Vector) cells. Left panel: representative photographs of cell colonies; right panel: bar graph summarizing the number of colonies per field (top: C42B; bottom: PC3) (**p < 0.01, ***p < 0.001, Student's t-test; n=6). (**D**) Cell viability assay of SIRT3 stably overexpressed (SIRT3) and control (Vector) cells (top: C42B; bottom: PC3. ***p < 0.001, two-way ANOVA, followed by post-hoc tests). (**E**) *In vivo* tumor formation assay of SIRT3 overexpressed vs. control vector transfected C42B prostate cancer cells. The tumor volumes (***p < 0.001, two-way ANOVA, followed by post-hoc tests) and tumor weights (***p < 0.001, Student's t test) between two groups were analyzed. Dissected tumors from mice at the end of the experiment were also photographed. Data of Fig D, E (tumor volume) are expressed as means ±SD. Data of rest figures are expressed as means ±SEM.

In order to explore the function of SIRT3 in prostate cancer, we first generated two SIRT3 stably overexpressed, castration-resistant prostate cancer (CRPC) cell lines that originally expressed low levels of SIRT3 (C42B-SIRT3 and PC3-SIRT3). Immunofluorescence microscopy and Western blot analysis confirmed that significantly higher level of SIRT3 was expressed in these cells than the control vector transfected counterparts (Figure 2A & 2B). Overexpression of SIRT3 markedly reduced colony formation and cell viability of prostate cancer cells *in vitro* (Figure 2C & 2D). Cell growth inhibition by SIRT3 was also observed in the three dimensional soft agar assay model (Figure S1A). *In vivo* subdermal tumor formation assay confirmed the inhibitory effect of SIRT3 in prostate cancer (Figure 2E, Figure S1B). Furthermore, the renal capsule model [14, 15] also showed that SIRT3 overexpression suppressed the prostate tumor formation (Figure S2). Together these results demonstrated that overexpression of SIRT3 suppressed prostate cancer cell growth *in vitro* and *in vivo*.

### Knockdown of SIRT3 accelerates prostate cancer cell proliferation *in vitro* and in vivo

**Figure 3:**
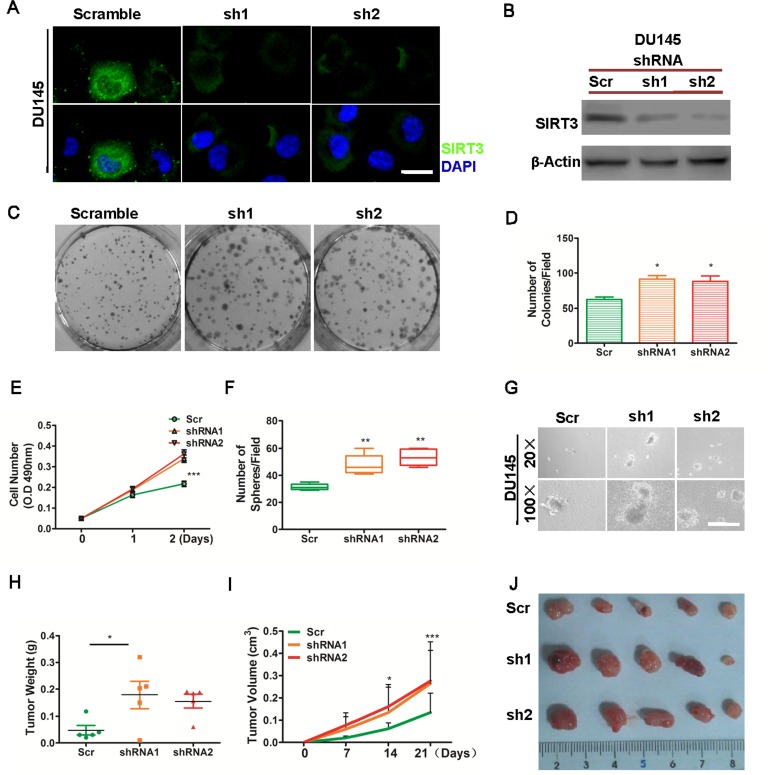
Knockdown of SIRT3 promotes prostate cancer proliferation DU145 cells stably transfected with sh-SIRT3 lentivirus (sh1 and sh2) or scrambled control virus (scramble) were subjected to various assays. (**A**) Immunofluorescence microscopy analysis of SIRT3 expression. SIRT3 (green) and nuclei (DAPI staining), Bar=20 μm. (**B**) Western blot analysis of SIRT3 expression. Beta-actin serves as loading control. (C-D) Clone formation assay. (**C**) Representative photographs of cell colonies; (**D**) Bar graph summarizing the number of colonies per field (*p < 0.05, Student's t test). (**E**) Cell viability assay (***p < 0.001, two-way ANOVA, followed by post-hoc tests). (F-G) Soft agar assay. The number of tumor spheres per field was summarized in (**F**) (Bar=100 μm, **p < 0.01, one-way ANOVA followed by Tukey's multiple comparison test) and representative images were shown with low (20X) and high (100X) magnifications in (**G**). (**H**-**J**) *In vivo* tumor formation assay of these cells. Tumor weights (*p < 0.05, one-way ANOVA followed by Tukey's multiple comparison test) and tumor volumes (*p < 0.05, ***p < 0.001, two-way ANOVA followed by post-hoc test among the three groups) were summarized and photographs of dissected tumors at the end of experiments were shown. Data of Fig E, I (tumor volume) are expressed as means ±SD. Data of rest figures are expressed as means ±SEM.

We then tested whether SIRT3 knockdown could affect prostate cancer cell growth. Using lentivirus containing two specific SIRT3 targeting shRNA sequences, we generated DU145-shSIRT3 cell lines. Immunofluorescence microscopy and Western blot confirmed the SIRT3 knockdown effect in these two cell lines (Figure 3A & 3B). Silencing of SIRT3 significantly promoted prostate cancer cell growth as determined by colony formation assay and cell viability assay (Figure 3C–3E). In addition, SIRT3 silencing also promoted cancer cell growth as shown in the three dimensional soft agar model (Figure 3F & 3G). Subcutaneous tumor formation assay by which tumor volume and dissected tumor weight was determined, clearly showed that knockdown of SIRT3 promoted prostate cancer growth *in vivo* (Figure 3H–3J).

### SIRT3 induces destruction of oncoprotein c-MYC in prostate cancer cells

**Figure 4:**
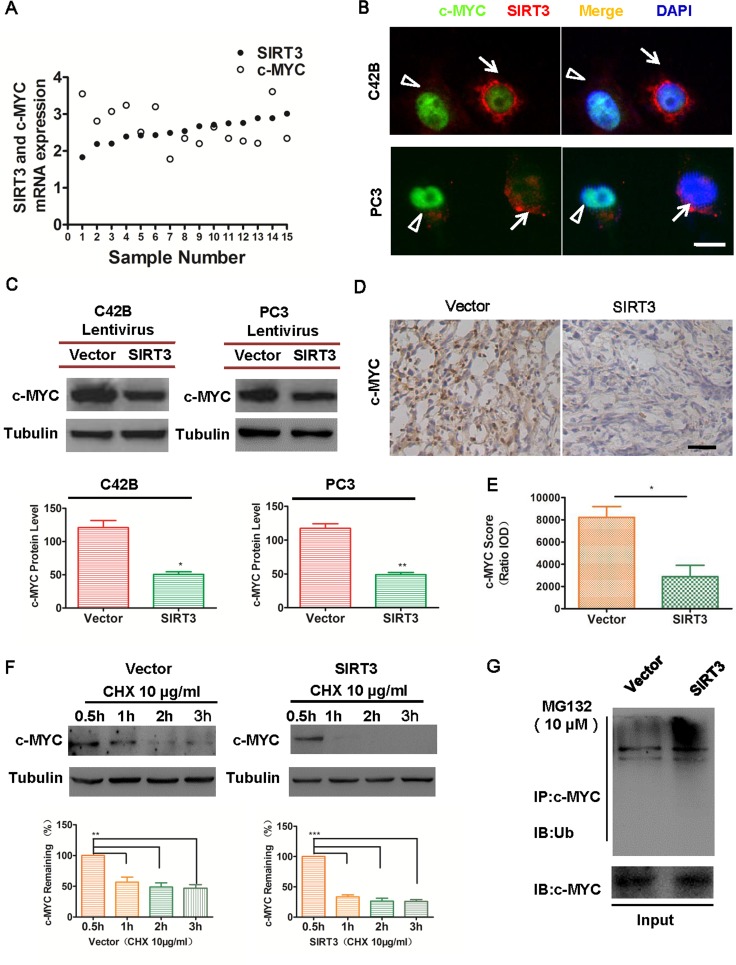
SIRT3 induces oncoprotein c-MYC destruction in prostate cancer cells (**A**) Relative mRNA expression levels of SIRT3 and c-MYC in prostate carcinoma (PCa) samples from selected *Oncomine* database (Luo2 Prostate Statistics, n=15). (**B**) Immunofluorescence microscopy of c-MYC and SIRT3 in prostate cancer cells. White arrows indicates cells with high SIRT3 expression and white triangle indicates cells with high c-MYC expression (Bar=10 μm). (**C**) Western blot and densitometry analysis of c-MYC expression between SIRT3 overexpressed group and control group in CRPC cell line C42B. (*p < 0.05, Student's t test). (**D**) Immunohistochemical microscopy analysis of c-MYC in the frozen section of tumors formed from renal capsule model. (**E**) Analysis of c-MYC staining intensity in (D). (Bar=100 μm, *p < 0.05, Student's t test). (**F**) Western blot analysis of the degradation level of c-MYC between SIRT3 overexpressed group and control group in C42B treated with cycloheximide (CHX, 10 μg/ml). (**p < 0.01, ***p < 0.001, one-way ANOVA followed by Tukey's multiple comparison test). (**G**) Immunoprecipitation assay analysis of the ubiquitination level of c-MYC between SIRT3 overexpressed group and control group in C42B treated with the proteasome inhibitor MG132 (10 μM) for 8 h. Data are expressed as means ±SEM.

To understand the molecular mechanism of SIRT3 in prostate cancer, we focused on specific oncoproteins. As an oncoprotein, c-MYC has been reported to maintain tumorigenicity and accelerate tumor growth in different cancer types [16–18]. Furthermore, c-MYC is found to be up-regulated in prostate cancer and highly correlated with the progression of the disease [19–21]. As SIRT3 effectively suppressed prostate cancer cell proliferation (Figure 2), we speculated that SIRT3 might function through the suppression of oncoprotein c-MYC. First, we examined datasets from *Oncomine* and found that in prostate cancer patients relatively higher SIRT3 mRNA expression level was correlated with lower c-MYC expression (Figure 4A). Moreover, prostate cancer cells with higher SIRT3 protein level expressed lower c-MYC level (Figure 4B). These results implicated an inverse relationship between the expressions of SIRT3 and c-MYC. We then examined the expression of c-MYC in SIRT3-overexpressed cancer cell lines and control cells. Indeed, overexpression of SIRT3 led to down-regulation of c-MYC both *in vitro* (Figure 4C) and *in vivo* (Figure 4D & 4E). Conversely, knockdown of SIRT3 led to the upregulation of c-MYC protein level (Figure S3A & S3B). These data demonstrated that SIRT3 could suppress specific oncoprotein c-MYC in prostate cancer.

To further elucidate the mechanism by which SIRT3 repressed c-MYC levels, we sought to test whether SIRT3 could affect the stability of c-MYC protein. Destruction of c-MYC was attributed to ubiquitin-mediated proteolysis in different cancers [22–24]. We examined SIRT3's role on the degradation and ubiquitination of c-MYC in prostate cancer. The half-life of c-MYC protein was significantly reduced in SIRT3 overexpressed prostate cancer cells, in comparison with the vector group (Figure 4F). In addition, c-MYC protein level was restored when cancer cells were treated with proteasome inhibitor MG132 (Figure S4A). These results indicated that SIRT3 might inhibit c-MYC by ubiquitin-mediated proteolysis. Indeed, the ubiquitination level of c-MYC protein was increased in SIRT3 overexpressed cancer cells (Figure 4G). It has been reported that c-MYC phosphorylation at T58 was required for ubiquitination and degradation by proteasome [19, 25]. We analyzed the phosphorylation of c-MYC at T58 and observed marked increase in two prostate cancer cell lines after SIRT3 overexpression (Figure S4B). Together these data demonstrated that SIRT3 induced oncoprotein c-MYC destruction by stimulating ubiquitin-mediated proteolysis in prostate cancer cells.

### SIRT3 destabilizes oncoprotein c-MYC level by regulating the PI3K-Akt pathway

**Figure 5:**
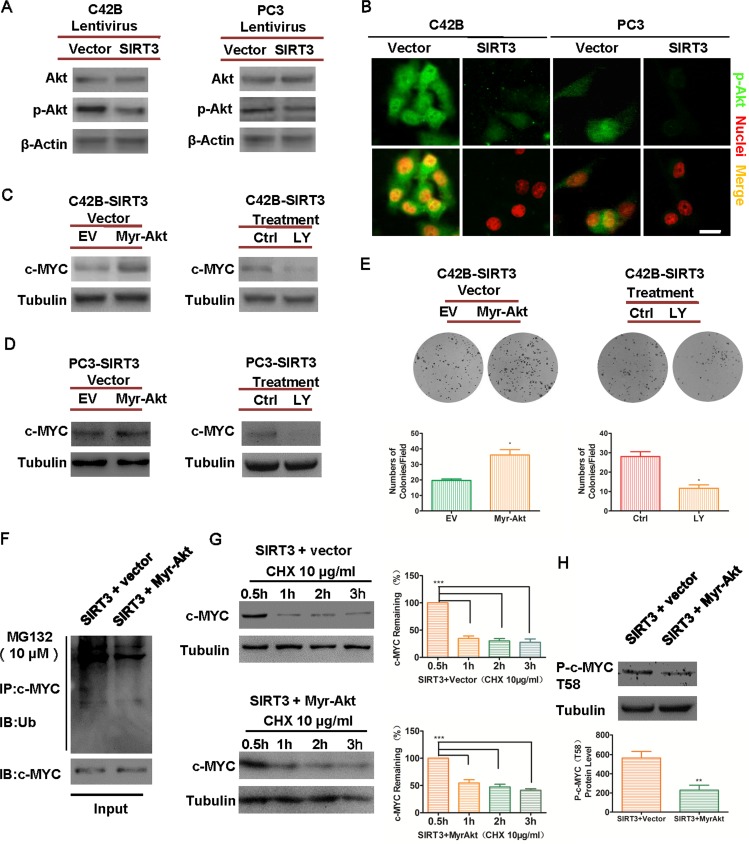
SIRT3 destabilizes oncoprotein c-MYC level by inhibiting PI3K/Akt pathway (**A**) Western blot analysis of phospho-Akt (Ser473) and Akt levels in SIRT3 overexpressed group and control (Vector) group of prostate cancer cells. (**B**) Immunofluorescence staining of p-Akt in SIRT3 overexpressed cells and Vector control cells (Bar =20μm). (**C**) and (**D**) Western blot analysis of c-MYC levels in SIRT3-overexpressed cells that were either transfected with Myr-Akt (vs. empty vector EV) or treated with PI3K-Akt pathway inhibitor LY294002 (LY 50 μM) vs. vehicle control (Ctrl). (**E**) Clone formation of the cancer cells mentioned in (C). (*p < 0.05, Student's t test). (**F**) Immunoprecipitation assay analysis of the ubiquitination level of c-MYC in SIRT3-overexpressing C42B cells that transfected with Myr-Akt (vs. empty vector EV). (**G**) Western blot analysis of the degradation level of c-MYC in SIRT3-overexpressing C42B cells that transfected with Myr-Akt (vs. empty vector EV). (***p < 0.001, one-way ANOVA followed by Tukey's multiple comparison test). (**H**) Western blot analysis and staining intensity analysis of p-c-MYC (T58) expression between SIRT3 overexpressed group and control group in C42B cell line. (**p < 0.01, Student's t test). Data are expressed as means ±SEM.

The next question was to determine the signaling pathway(s) that underlay the regulation of c-MYC stability by SIRT3 in prostate cancers. Akt/mTOR pathway was highly activated in CRPC [26–28] and p-Akt was reported to target and stimulate the expression of tumorigenic factors like c-MYC [16], SOX2 [29, 30], OCT4 [31] and eventually robust tumor growth. SIRT3 has been reported to repress the activation of Akt in cardiac hypertrophy [32, 33], we therefore hypothesized that SIRT3 could inhibit oncoproteins via the Akt pathway in prostate cancer. Indeed, we found that the activation of Akt (p-Akt Ser473) was attenuated by SIRT3 overexpression both *in vitro* and *in vivo* (Figure 5A & 5B, and Figure S5). Conversely, p-Akt Ser473 was enhanced by SIRT3 silencing in prostate cancer cells (Figure S6A & S6B). These results indicated that SIRT3 suppressed the activation of Akt in prostate cancer cells.

To further investigate whether SIRT3 destructed oncoprotein c-MYC through the Akt pathway, we performed rescue experiments. In SIRT3 overexpressed prostate cancer cells we transfected a constitutively active, myristoylated form of Akt (Myr-Akt), or applied LY294002, a selective inhibitor of the PI3K/Akt pathway to inhibit p-Akt Ser473 signals [34] (Figure S7). The expression of c-MYC originally blocked by the overexpression of SIRT3 was up-regulated by the activation of Akt, and conversely c-MYC level could be further reduced by direct inhibition of PI3K/Akt pathway (Figure 5C & 5D). In addition, forced expression of constitutively active Akt attenuated the inhibitory effect of SIRT3 showing up-regulation of p-Akt Ser473 levels and increased colony formation, whereas inhibition of Akt signaling suppressed prostate cancer cell growth (Figures 5E & S8). Mechanistically, the ubiquitination level of c-MYC was reduced (Figure 5F) and degradation of c-MYC was attenuated (Figure 5G) by the activation of Akt in SIRT3 overexpressed prostate cancer cells. In addition, c-MYC phosphorylation is required for its ubiquitin-mediated degradation [19, 35]. We also noticed that the phosphorylation level at c-MYC T58 decreased upon Akt activation, corresponding to the lower ubiquitination level of c-MYC (Figure 5H).

### SIRT3 suppresses PI3K/Akt pathway through down-regulating ROS level

**Figure 6:**
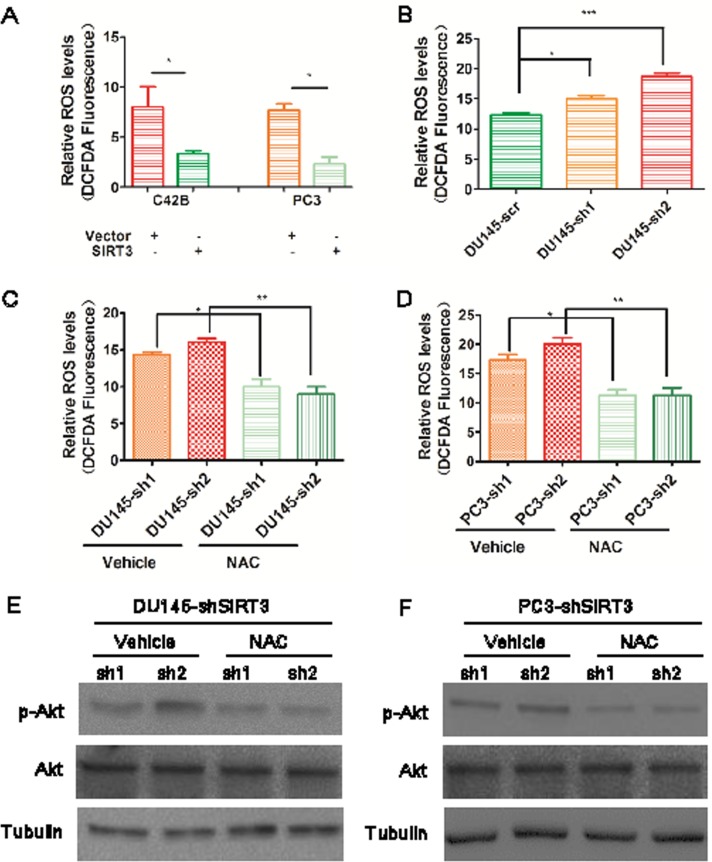
SIRT3 suppresses PI3K-Akt pathway through down-regulating ROS levels (**A**) and (**B**) Quantitative analysis of the ROS level in different prostate cancer cells up- or down-regulated with SIRT3 (*p < 0.05, **p < 0.01, ***p < 0.001, one-way ANOVA followed by Tukey's multiple comparison test). (**C**) and (**D**) Quantitative analysis of the ROS level in different SIRT3 knock out cancer cells treated with NAC (10μM) and vehicle control (*p < 0.05, **p < 0.01, one-way ANOVA followed by Tukey's multiple comparison test). (**E**) and (**F**) Western blot analysis illustrated the p-Akt (Ser473) protein levels after these treatments. Data are expressed as means ±SEM.

SIRT3 is a mitochrondria-located antioxidants trigger [9, 12] and ROS can activate the PI3K/Akt pathway [26, 36]. SIRT3 has also been reported to repress the activation of Akt in cardiac hypertrophy through ROS and relieve the disease symptoms [32, 33]. We hypothesized that SIRT3 might function by regulating the Akt pathway through ROS in prostate cancer. To test this, we measured ROS levels in three different CRPC lines. Overexpression of SIRT3 significantly suppressed the basal ROS levels (Figure 6A) while knockdown of SIRT3 increased ROS levels (Figure 6B). The activation of Akt (p-Akt Ser473) was also enhanced when SIRT3 was blocked (Figure S6). To explore whether SIRT3 could inhibit Akt phosphorylation at Ser473 by blocking ROS, we used antioxidant N-acetyl cysteine (NAC) to suppress the high ROS level in SIRT3 knockdown cancer cells (Figure 6C & 6D) and measured the p-Akt(Ser473) level concurrently. The p-Akt (Ser473) level was evidently reduced in the presence of NAC in SIRT3 silenced prostate cancer cells (Figure 6E & 6F). These data suggested that SIRT3 inhibited the activity of PI3K/Akt pathway by suppressing ROS in prostate cancer cells.

## DISCUSSION

**Figure 7:**
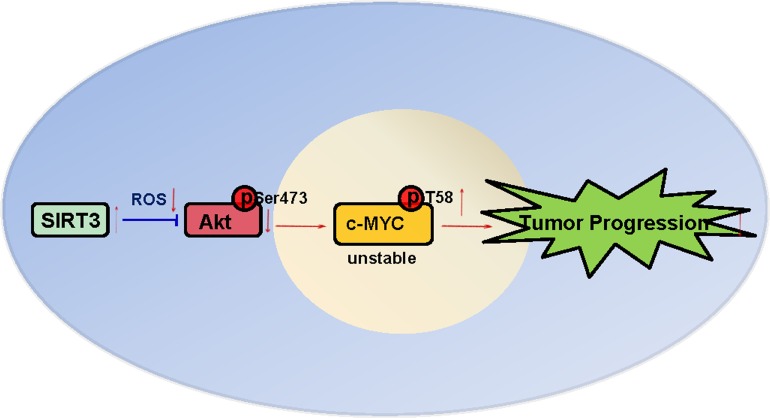
A model summarizing the suppression of tumor progression by SIRT3 in prostate cancer as illustrated in this study

In this study, we have demonstrated for the first time that SIRT3 is moderately down-regulated in prostate cancers and patients with high SIRT3 copy number exhibit a significantly longer overall survival compared to those with low SIRT3 copy number. We have also shown that SIRT3 suppresses prostate cancer growth both *in vitro* and *in vivo* by inhibiting the activation of PI3K/Akt, thereby leading to the destruction of oncoprotein c-MYC. We show a SIRT3-Akt-c-Myc signaling axis that underlies the progression of prostate cancer (Figure 7), and suggest that the molecular and pathological determination of SIRT3 may be used in prostate cancer diagnosis/prognosis. Strategies targeting this pathway may provide novel therapeutic options for prostate cancer.

It is crucial to point out that SIRT3 suppresses prostate cancer progression through the inhibition of PI3K/Akt pathway and eventually down-regulation and destruction of oncoprotein c-MYC. Such molecular mechanism underlying the suppression of tumor by SIRT3 has not been reported before. SIRT3 has been regarded as an aging-related protein and most studies of SIRT3 have focused on longevity [37–39]. SIRT3 was first reported as a tumor suppressor in breast carcinoma [12]. This mitochrondrial tumor suppressor was later found to decrease hypoxia-inducible factor 1α (Hif1α) and genomic instability, which led to cellular metabolic reprogramming and eventually limited carcinogenesis [13, 40]. However, whether SIRT3 played a role in regulating proto-oncogenes had remained unknown. Oncoproteins like c-MYC robustly drive tumor development and are key players in various types of cancers including prostate cancer [22, 24]. Herein we established a link between SIRT3 and c-MYC, which was connected by PI3K/Akt pathway. It has been shown that key signaling transduction pathways such as the Akt/mTOR pathway affect cancer cell survival and are highly activated in prostate cancer [26–28]. In addition, the activation of oncoprotein such as c-MYC is regulated by Akt pathway [22, 25]. Targeting c-MYC could put a brake on the rapid growth of cancer [35, 41, 42], but c-MYC is currently undruggable. Fortunately the fast rate of c-MYC protein degradation (half-life of ~30 min in rapidly dividing cells) allows tight regulation on c-MYC activity [43]. We here showed up- or down-regulation of SIRT3 affected c-MYC level and stability, which was mediated by the PI3K/Akt pathway. By resuming the activation of PI3K/Akt pathway, phosphorylation level at T58 of c-MYC decreased, leading to the reduction of the ubiquitin-mediated proteolysis of c-MYC. Our data suggest that controlling the activity of SIRT3 might be an alternative approach to manage c-MYC in cancer cells.

The Sirtuin family proteins play diverse roles in cancer development [44]. In addition to destabilizing c-MYC as reported herein, SIRT3 could function as a tumor suppressor possibly via its ability to deacetylate the proto-oncogene Skp2 [45]. Skp2 is overexpressed in a wide range of cancers including prostate cancer. In this regard, developing small molecules to activate SIRT3 might be a sound strategy for prostate cancer treatment. Our group has recently shown that a small molecule drug adjudin could efficiently up-regulate SIRT3 in normal cells [46]. Adjudin was also reported to be a potential anti-cancer drug as it targets proliferating cells [47]. To our best knowledge, no clinical trials have been reported using SIRT3 activator to treat cancer. As adjudin is generally regarded as a safe molecule [48, 49], future experiments to test the effectiveness of adjudin in prostate cancer and how it affects the Akt and c-MYC are warranted. On the other hand, structure-based design of SIRT3 regulatory molecules should also be actively pursued and investigated for the treatment of prostate cancers.

## MATERIALS AND METHODS

### Prostate cancer cell lines, plasmids and transfection

Human prostate cancer cell lines DU145, PC3 and C42B were purchased from Shanghai Cell Collection (Shanghai, China) and maintained in basic Dulbecco's modified Eagle's medium (DMEM, GIBCO) supplemented with 10% heat-inactivated fetal bovine serum (FBS, GIBCO). All cell lines were grown at 37°C in a humidified 5% CO_2_ atmosphere. Full-length SIRT3 (plasmid #13814) and Myr-Akt (plasmid # 9005) plasmids were purchased from Addgene (www.addgene.org). Transfections were performed using jetPRIME (Polyplus) according to the manufacturer's instructions.

### Construction lentiviral vectors and lentivirus production

For lentivirus-based overexpression system, full-length human SIRT3 from Addgene (plasmid #13814) was cloned into a lentiviral vector under the control of CMV promoter. In brief, PCR was performed to obtain the SIRT3 cDNA using the primers 5′-CCGGAATTCGCCACCATGGCGTTCTGGGGTTGGCGCGCCGCGGCAGC-3′ and 5′-CTAGTCTAGACTATTTGTCTGGTCCATCAAGCTTCCCAGTTTCCCG-3′. The PCR product was purified and subcloned into EcoRI and XhoI sites of the lentiviral pLVX-Neo-IRES vector (Biowit Company, www.biowit.com.cn). The pLVX-Neo-IRES vector without any sequence in MCS (multiple clone sites) was used as the control plasmid.

For lentivirus-based shRNA knockdown system, the specific target sequences of SIRT3 (sh1: 5′-CAACGTCACTCACTACTTT-3′; sh2: 5′-GGGTGCTTCAAGTGTTGTT-3′) were cloned into the lentiviral shRNA vector under the control of U6 promoter. GV298 containing scrambled sequence was used as the control plasmid

Lentivirus was produced by transfecting the packaging plasmids pCMV-dR8.74, pMD2.G (Addgene) as well as the transfer lentiviral plasmids into HEK-293T cells with calcium phosphate precipitation method. Medium containing lentivirus was harvested and filtered after 72h and 96 h of transfection. The lentivirus was concentrated from supernatants by ultracentrifugation and stocked at −80°C.

### Lentiviral infection

Stable cell lines were generated by infection of cells with lentivirus, which was carried out in 24-well plate with serum-free DMEM medium. C42B, PC3 cells were transduced with lenti-SIRT3 at the infection MOI ≥90, and DU145, PC3 cells were transduced with lenti-sh-SIRT3 at the infection MOI ≥90 at 37°C with 8μg/ml polybrene for 24 h. Then culture medium with 10% FBS was replaced and cells were continuously cultured for 4 to 6 days followed by selection with G418 (Invitrogen) at500μg/ml.

### Colony formation and soft agar assay

Clonogenic survival assays were performed by plating approximately 500-1,000 cells in 6-well culture dishes. In some experiments, cells were treated with vehicle (control) or treated with drugs. Cells were then fixed with 4% paraformaldehyde, stained with crystal violet solution and formed colonies (≥50 cells) were visually counted. Soft agar assay was performed in a six-well culture plate that was coated with 2 ml bottom agar mixture (DMEM with 10% FBS, 0.6% agar). After the bottom layer was solidified, 2 ml top agar-medium mixture (DMEM with 10% FBS, 0.3% agar) containing 10,000 cells were added, and the plate was incubated at 37°C for 2-3 weeks. Then the colonies were counted.

### Cell proliferation assay

Cell viability was assessed by using Cell Titer 96® Aqueous One Solution Cell proliferation Assay kit (Promega, USA). This test is based on the change of MTS (3-(4,5-dimethylthiazol-2-yl)-5-(3-carboxymethoxyphenyl)-2(4-sulfophenyl)-2H tetrazolium), into a formazan product by NADPH or NADP in metabolically active cells. Medium was removed, washed with PBS. Then 20μl/well of Cell Titer 96® Aqueous One Solution was added and a 100μl/well of incubated solution medium was transferred to 96-well plate. After an hour, the optical density was read at 490 nm in a microplate reader (BioTek).

### Immunohistochemistry and immunofluorescence microscopy

To examine the staining pattern of various target proteins in prostate cancer cells, the fixed preparations were first permeablized in 0.5% Triton X-100 for 30 min, blocked with 10% normal goat serum at room temperature for 30 min, and then incubated with SIRT3(Cell Signaling), p-Akt (Ser473) (Cell Signaling), c-MYC (Epitomics) overnight at 4°C. For immunofluorescence analyses, Texas Red- or FITC-conjugated secondary antibodies (Jackson ImmunoResearch) were then used to reveal the labeling patterns. For immunohistochemistry analyses, HRP-conjugated secondary antibody (Jackson ImmunoResearch) was used. Negative controls were performed by skipping the primary antibody step. The labeled tissue and cells were visualized under a microscope (Leica DFC420C) and images were processed using Adobe Photoshop software.

### Western blot and immunoprecipitation assay

Cells were lysed in RIPA buffer (Millipore) supplemented with Complete Protease Inhibitor Cocktail (Roche), 2 mM PMSF, and 0.1% SDS. The protein concentration was measured using the BCA assay kit (Thermo Scientific). Total protein (~30 μg) was separated by 10% SDS-PAGE and then transferred to 0.45 μm nitrocellulose membrane (Millipore). The membrane was blocked with TBST containing 5% non-fat milk, incubated with SIRT3 (Cell Signaling), Akt (Cell Signaling), p-Akt Ser473 (Cell Signaling), c-MYC (Epitomics&Santa Cruz), p-c-MYC(T58) (Epitomics), Ubiquitin (Santa Cruz), β-tubulin (Epitomics), β-actin (Epitomics) at 4°C overnight and then hybridized with appropriate HRP-conjugated secondary antibody at room temperature for 1 h. Protein signals were visualized using ECL detection system (Thermo Scientific).

For immunoprecipitation, 150-500 μg lysates prepared as above were incubated on ice with 4-10 μg of the appropriate antibodies and 20 μl protein G-sepharose beads (Roche) overnight. Immuno-complexes were washed 4-5 times with PBS (Invitrogen) before immunoblotted with the indicated antibodies.

### Analysis of ROS production in cancer cells

The intracellular ROS level was measured by 2h,7′-dichlorofluorescein diacetate (DCFH-DA, Beyotime). In the presence of ROS, DCFH is converted into the highly fluorescent 2i,7′7acchlorofluorescein (DCF), which produces bright green fluorescence. Briefly, different prostate cells were cultured in a 24-well plate. After receiving various treatments, cells were washed twice in PBS and incubated with 10 μM DCFH-DA at 37°C in a humidified atmosphere of 95% air and 5% CO2. After 30 min, the extracellular ROS dye was washed away with DMEM without serum and the 24-well plate was then put in a microplate reader (BioTek) to measure the level of fluorescence. The excitation and emission wavelengths used were 482 nm and 535 nm, respectively.

### Tumor formation assay *in vivo*

Male Balb/c nude mice of 3-5 weeks age (purchased from SLAC Laboratory, Shanghai) were maintained in pathogen-free conditions at animal facility of School of Biomedical Engineering, Shanghai JiaoTong University. For measuring the tumor formation ability of the various groups of prostate cancer cells, these cells were resuspended in serum-free medium and mixed with Matrigel at the ratio of 1:1, followed by subcutaneously injection into Balb/c nude mice (20,000cells per mouse). For measuring the tumor formation ability with renal capsule model, SIRT3 overexpressed group and control group of cancer cells combined with murine urogenital sinus mesenchyme (UGSM) cells (1:1) were mixed with rat-tail collagen and grafted under kidney capsule of Balb/c nude mice. Tumor formation was evaluated once every three days after injection by palpation of injection sites. Between2 weeks to 1 month, animals were killed and the tumor weights were measured. All studies were approved by the Institutional Animal Care and Use Committee, Shanghai Jiao Tong University, and all animals were treated in accordance with the institutional guidelines.

### Statistical methods

The GraphPad Prism software was used in data processing and statistical analysis of significance. Data are presented as mean±SEM or mean±SD (If mentioned). Students' test was used to compare two groups (p < 0.05 was considered significant) and ANOVA with Tukey post-hoc test was used to compare three or more groups (p < 0.05 was considered significant).

## SUPPLEMENTARY MATERIAL FIGURES AND TABLE


