# Supplementary material for: SIRT3 inhibits prostate cancer by destabilizing oncoprotein c-MYC through regulation of the PI3K/Akt pathway
Source: Oncotarget. 2015 Jul 3;6(28):26494–507. doi: 10.18632/oncotarget.4764 (PMC4694917; doi:10.18632/oncotarget.4764)
Supplement: Supplementary file 1 [file oncotarget-06-26494-s001.pdf]

SIRT3 inhibits prostate cancer by destabilizing oncoprotein c-MYC through regulation of the PI3K/Akt pathway

Supplementary Material

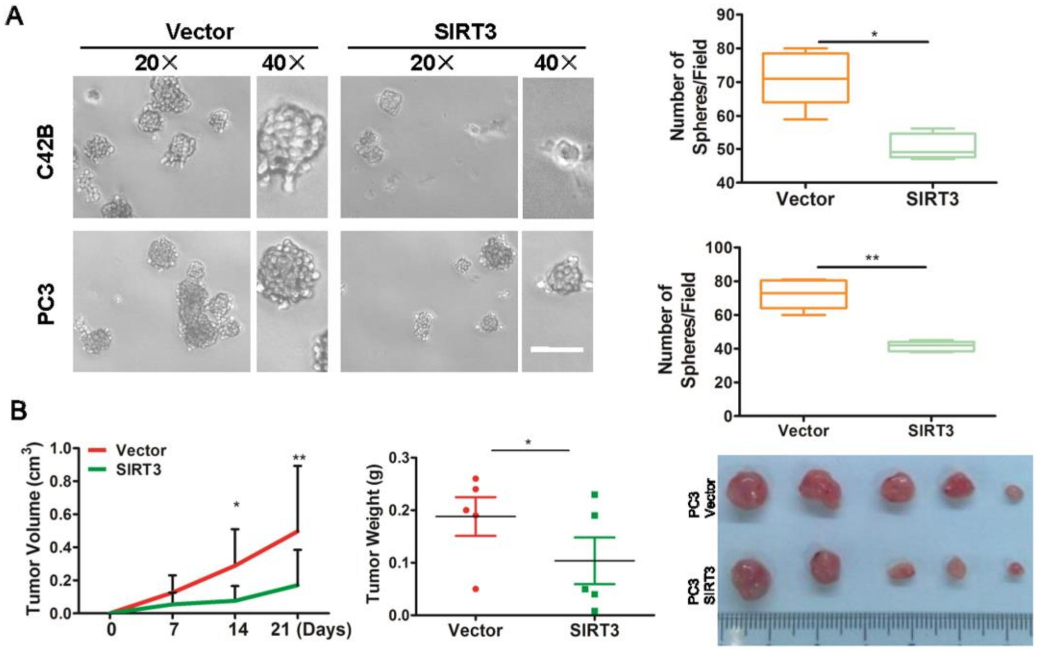

Fig.S.1 Overexpression of SIRT3 blocks prostate cancer cell proliferation. (A) Soft agar assay of stably overexpressed (SIRT3) and control (Vector) cells. Representative images were shown with low (20X) and high (40X) magnifications (left panel); the number of tumor spheres per field was summarized (right panel. top: C42B cells; bottom: PC3 cells). (Bar=100 μm, *$p < 0.05$, Student's t test). (B) *In vivo* tumor formation assay of SIRT3 overexpressed vs. control vector transfected PC3 prostate cancer cells. The tumor volumes (*$p < 0.05$, **$p < 0.01$, two-way ANOVA, followed by post-hoc tests) and tumor weights (*$p < 0.05$, Student's t test) between two groups were analyzed. Dissected tumors from mice at the end of the experiment were also photographed. Data of Fig B (tumor volume) is expressed as means ±SD. Data of rest are expressed as means ±SEM.

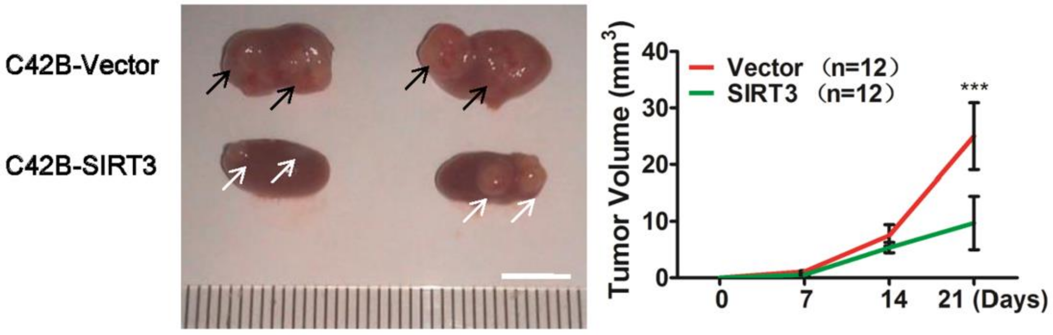

Fig.S.2 *In vivo* tumor formation assay in renal capsule model of SIRT3 overexpressed vs. control vector transfected C42B prostate cancer cells. White arrows indicate tumor formed from SIRT3 overexpressing cells and black arrows indicate tumor formed from control cells. The tumor volumes between two groups were analyzed (***$p < 0.001$, two-way ANOVA, followed by post-hoc tests). Dissected tumors with kidneys from mice at the end of the experiment were also photographed. Data is expressed as means ±SD.

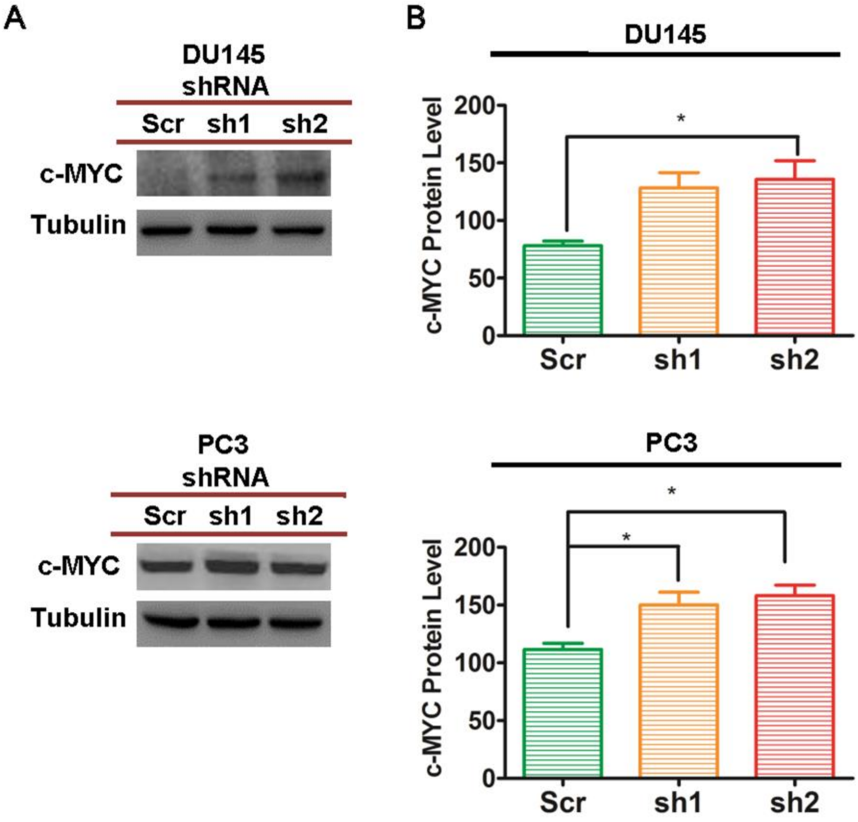

S.Fig.3 Knockdown of SIRT3 promotes c-MYC protein level in prostate cancer cells. (A) Western blot of c-MYC expression in SIRT3 knockdown prostate cancer cells. (B) Analysis of c-MYC staining intensity in (A). (*$p < 0.05$, one-way ANOVA followed by Tukey's multiple comparison test). Data are expressed as means ±SEM.

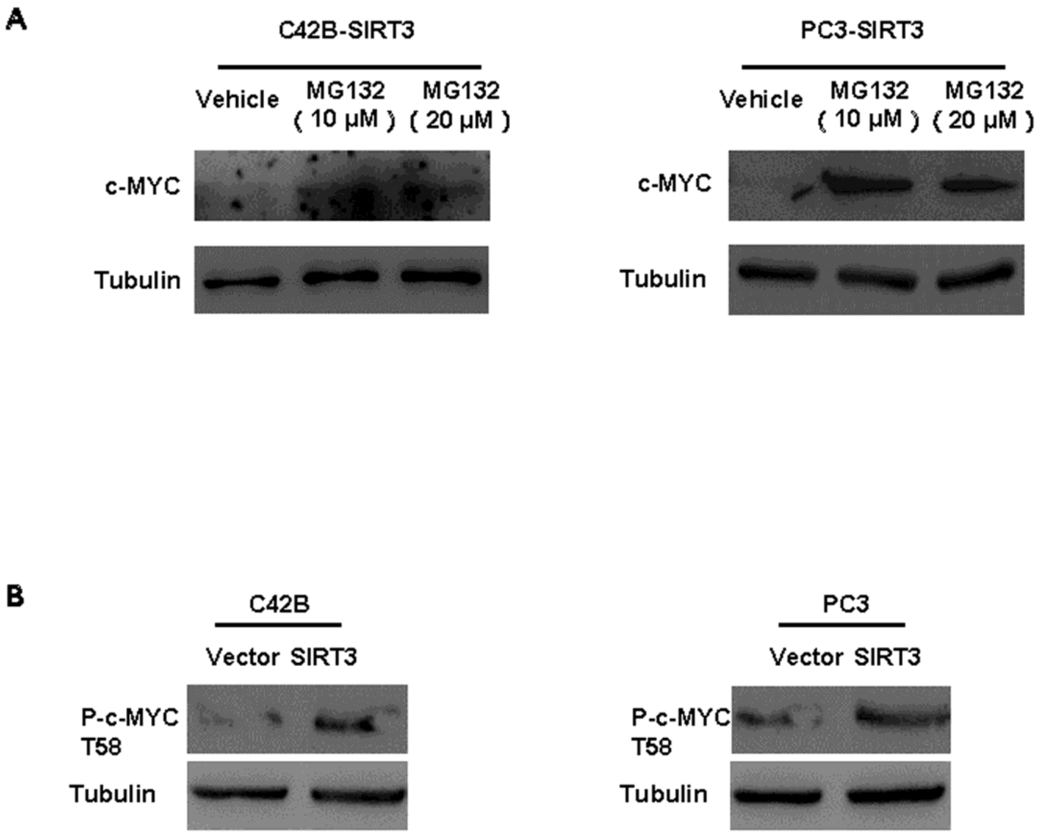

S.Fig.4 SIRT3 induces oncoprotein c-MYC destruction and promotes P-c-MYC (T58) level in prostate cancer cells. (A) C42B-SIRT3 and PC3-SIRT3 cancer cell lines were treated with proteasome inhibitor MG132 for 8 hours (B) Western blot analysis and staining intensity analysis of P-c-MYC (T58) expression between SIRT3 overexpressed group and control group in CRPC cell line C42B and PC3.

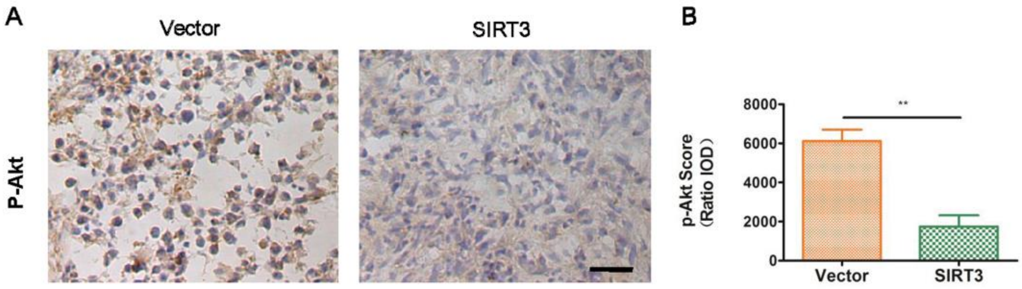

S.Fig.5 SIRT3 inhibits the activation of Akt pathway. (A) Immunohistochemical microscopy analysis of p-Akt Ser473 in the frozen section of tumors generated from mouse renal capsule. (B) Analysis of p-Akt Ser473 staining intensity in (A). (Bar=100μm, **$p < 0.01$, Student's t test).

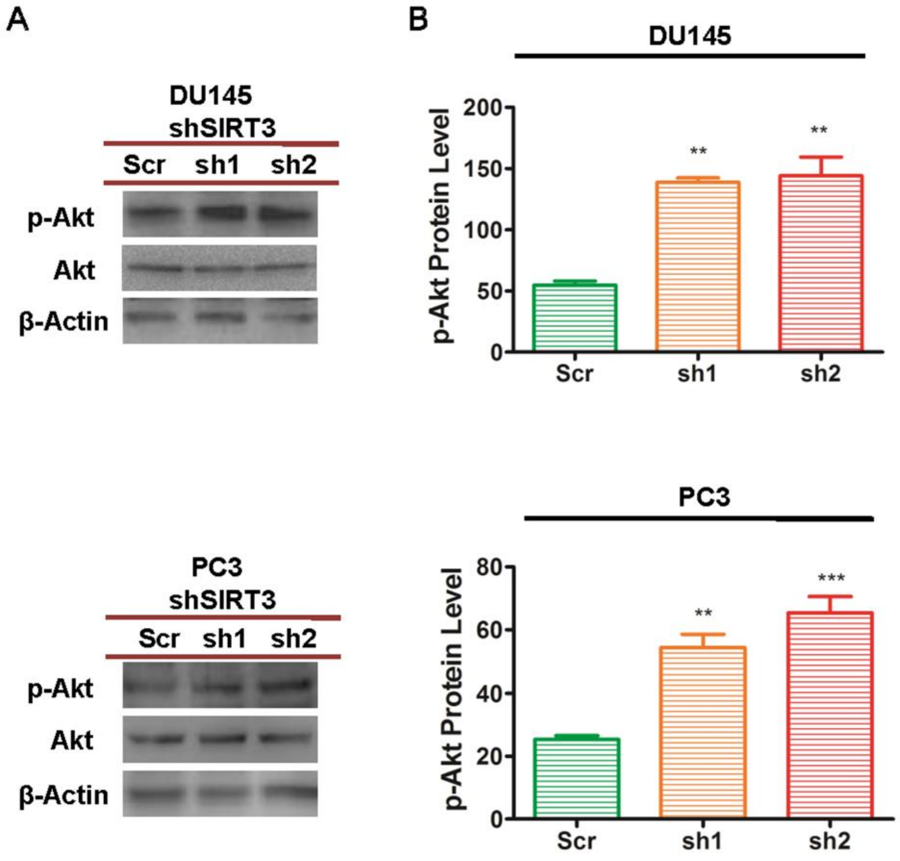

S.Fig.6 Knockdown of SIRT3 promotes the activation of PI3K-Akt pathway. (A) Western blot of p-Akt (Ser473) expression in SIRT3 knockdown prostate cancer cells. (B) Analysis of p-Akt (Ser473) staining intensity in (A). (**$p < 0.01$, ***$p < 0.001$, one-way ANOVA followed by Tukey's multiple comparison test). Data are expressed as means ±SEM.

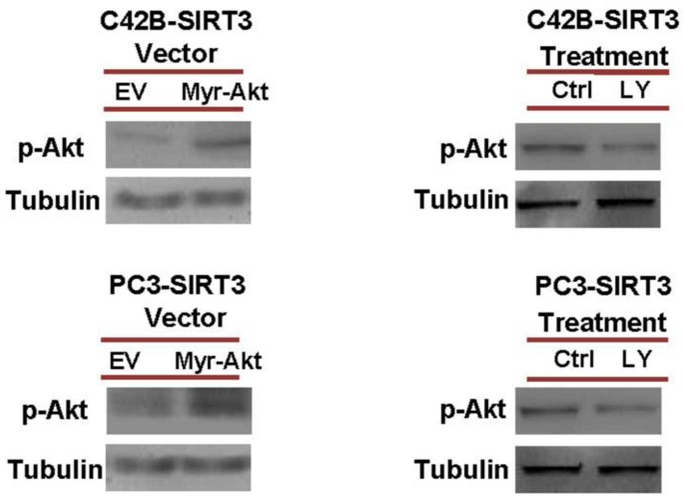

S.Fig.7 Activation and suppression level of PI3K-Akt pathway. Rescue experiments with a constitutively active myristoylated form of Akt (Myr-Akt) and an empty vector (EV) as control in SIRT3 overexpressed cancer cells. Reversely, selective PI3K-Akt pathway inhibitor LY294002 (LY, 50 μM) was used to inhibit Akt activation with equivalent concentration of DMSO as vehicle control (Ctrl) in SIRT3 overexpressed cancer cells. Western blot analysis illustrated the p-Akt protein levels after these treatments.

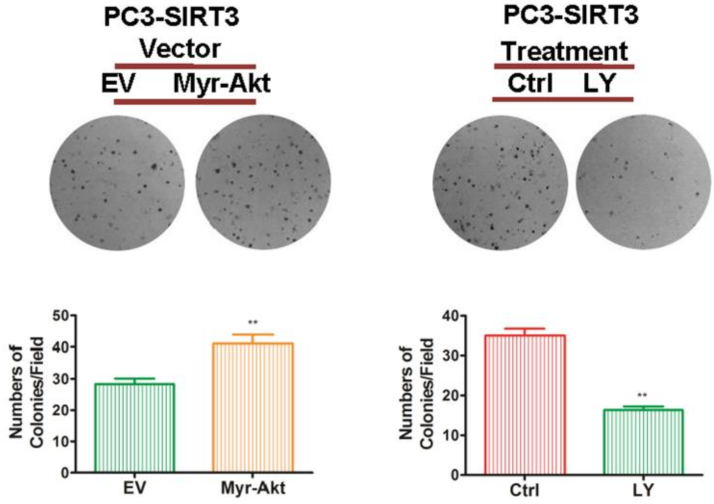

S.Fig.8 The constitutively activation of PI3K-Akt pathway promotes prostate cancer cell growth. Clone formation of the cancer cells mentioned in Figure5. (**$p < 0.01$, Student's t test). Data are expressed as means ±SEM.

Supplementary Table 1.

SIRT3 expression in carcinoma of the prostate according to patient's age and histopathologic characteristics

Variable	SIRT3 IHC			Total
	High	Medium	Low	
Patient's age(y)				
≤ 55	4/12 33%	3/12 25%	5/12 42%	12
> 55	20/97 21%	15/97 15%	62/97 64%	97
Tumor type				
Carcinoma				
Tumor grade				
Benign	18/32 56%	4/32 13%	9/32 28%	32
Gleason Score1-6	13/47 28%	11/47 23%	23/47 49%	47
Gleason Score7-9	3/27 11%	4/27 15%	20/27 74%	27
	$p<0.01$		$p<0.01$	
(3 undefined)				

Tumor staining intensity was scored as low (<10% positive), medium (10%-50% positive) and high (>50% positive) cytoplasmic staining depending on the percentage of positive cells. (chi-test)
